# Taxane-cisplatin-fluorouracil as induction chemotherapy for advanced head and neck cancer: a meta-analysis of the 5-year efficacy and safety

**DOI:** 10.1186/s40064-015-0988-5

**Published:** 2015-05-01

**Authors:** Xu Qian, Chenming Ma, Thomas K Hoffmann, Andreas M Kaufmann, Andreas E Albers

**Affiliations:** Department of Otolaryngology, Head and Neck Surgery, Charité-Universitätsmedizin Berlin, Campus Benjamin Franklin, Hindenburgdamm 30, 12200 Berlin, Germany; Department of Histology and Embryology, Zhejiang Provincial Key Laboratory of Medical Genetics, Wenzhou medical University, Wenzhou, China; Department of Otolaryngology, Head and Neck Surgery, University of Ulm, Ulm, Germany; Clinic for Gynecology, Charité-Universitätsmedizin Berlin, Campus Mitte and Benjamin Franklin, Berlin, Germany

**Keywords:** Head and neck carcinoma, Induction chemotherapy, Docetaxel, Cisplatin, Fluoruracil, Survival

## Abstract

**Background:**

The objective of this study was to compare the efficacy and safety of taxane (docetaxel or paclitaxel), cisplatin, and fluorouracil (Tax-PF) with cisplatin plus fluorouracil (PF) regimen by a meta-analysis of data retrieved from the literature.

**Methods:**

Seven randomized clinical trials were identified, which included patients with advanced head and neck cancer who underwent induction chemotherapy with either a Tax-PF or PF protocol. The outcomes included the 3-year and 5-year overall survival (OS) and progression-free survival (PFS), overall response rate (ORR) and different types of adverse events.

**Results:**

The 3-year OS rate (HR: 1.14; 95% CI: 1.03 to 1.25; *P* = 0.008), 3-year PFS rate (HR: 1.24; 95% CI: 1.08 to 1.43; *P* = 0.002), 5-year OS rate (HR: 1.30; 95% CI, 1.09 to 1.55;*P* = 0.003), 5-year PFS rate (HR: 1.39; 95% CI, 1.14 to 1.70; *P* = 0.001) and ORR to chemotherapy (OR 1.66; 95% CI, 1.35 to 2.05; *P* < 0.001) of the patients in the Tax-PF group were statistically superior to those in the PF group. In terms of toxicities, the incidence of febrile neutropenia (OR 2.36; 95% CI, 1.62 to 3.46; *P* < 0.001), alopecia (OR 8.22; 95% CI, 3.99 to 16.92; *P* < 0.001), diarrhea (OR 1.57; 95% CI, 1.05 to 2.36; *P* = 0.03) and leukopenia (OR 2.79; 95% CI, 1.86 to 4.21; *P* < 0.001) was higher in the Tax-PF group.

**Conclusion:**

The Tax-PF induction chemotherapy improved PFS and OS, and the ORR was better as compared to PF-based therapy regimens at the cost of a higher incidence of adverse events.

**Electronic supplementary material:**

The online version of this article (doi:10.1186/s40064-015-0988-5) contains supplementary material, which is available to authorized users.

## Introduction

Head and neck squamous cell carcinoma is one of commonest malignant tumors, frequently diagnosed in an unresectable advanced stage (Siegel et al. 2014). A meta-analysis on chemotherapy in head and neck cancer (MACH-NC) has demonstrated that concomitant chemoradio-therapy using traditional cisplatin and fluorouracil (PF) regimen improved the survival in patients with distant metastases and should be regarded as the principal treatment (Pignon et al. 2009). Nevertheless, investigators are continuously evaluating new regimens in the induction setting to improve ORR, PFS and OS. Among agents introduced in the 1990s, taxanes have shown great promise for the treatment of head and neck squamous cell carcinoma (HNSCC) (Schrijvers and Vermorken 2000). The clinical efficacy of induction chemotherapy using a PF regimen doubled while a three-drug combination of taxane (docetaxel or paclitaxel), cisplatin, and fluorouracil (Tax-PF) is still undergoing evaluation in several randomized controlled trials (RCTs) with varying results (Blanchard et al. 2013; Forastiere et al. 2013; Tural and Kilickap 2014).

This meta-analysis was conducted to review all eligible RCTs comparing combined therapy with or without taxanes, with the aim of investigating whether Tax-PF therapy is more efficient than PF therapy for advanced head and neck cancer while adverse effects are still tolerable.

## Materials and methods

### Literature search

PubMed, Embase, SpringerLink, MEDLINE, the Cochrane Library and the American Society of Clinical Oncology (ASCO) Annual Meeting and ASCO virtual meeting databases were searched for entries until June 2014, using the following search keywords: “randomized”, “head and neck cancer”, “HNSCC”, “induction chemotherapy” and “taxanes or docetaxel or paclitaxel”. Articles and general reviews of this topic were examined and excluded manually.

### Study selection

**Figure 1:**
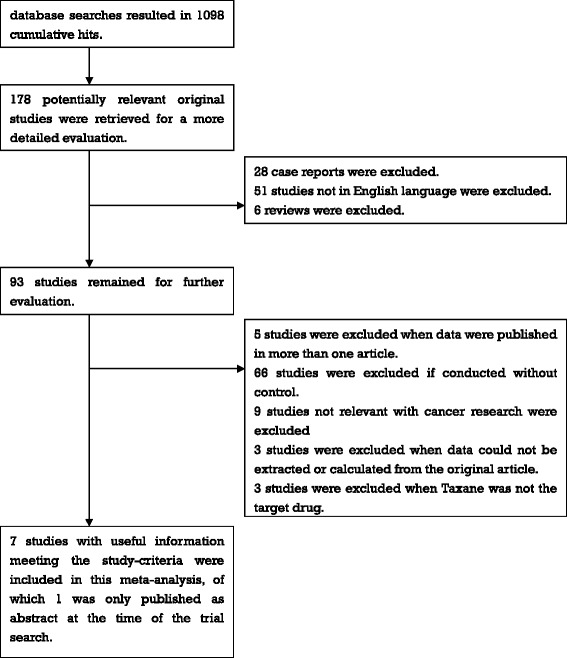
Flow-chart for identification of eligible studies.

Clinical trials that fulfilled the following criteria were included in the study: (1) prospective RCT; (2) original articles that include a censored number of patients or Kaplan–Meier-curves; (3) studies that analyzed combined therapy regimen with taxanes versus without taxanes; (4) comparisons of combined Tax-PF induction chemotherapy to regimen with traditional PF double-chemotherapy (Figure 1).

To limit publication bias, only published data were included. Studies containing one of the following criteria (1) phase I clinical trial; (2) retrospective trial; (3) any review, comment, and case report were excluded from the analysis.

### Data extraction and study quality

Studies were extracted from the databases by two of the authors separately. Next, between the authors a consensus was achieved that the data from the chosen publications met the inclusion criteria. Then the following information were extracted from each study, however some articles did not contain all of the following information: first author, publication year, country of patient’s origin, treatment regimen, patient number, age, gender, treatment line, ECOG (Eastern Cooperative Oncology Group) performance status (PS) or WHO status or Karnofsky status (KPS), median overall survival (OS), progression-free survival (PFS), overall response rate (ORR), adverse events (AEs), specific grade 3–4 toxicity data and general symptoms (alopecia, infection, and asthenia), hematological system syndrome (neutropenia, thrombocytopenia, and anemia), digestive system syndrome (nausea/vomiting, diarrhea, and anorexia), and neuropathy. In studies where the log hazard ratio (HR) and its variance was not explicitly presented the method described by Parmar et al. was applied to extract estimates of these statistics (Parmar et al. 1998). In those instances where studies contained overlapping sets of patients, the longest follow-up or the largest number of events was selected. The methodological quality of the reports integrated in this meta-analysis was evaluated using the Jadad composite scale (Jadad et al. 1996; Moher et al. 1998). A general quality score was applied to each study as follows: 0 (non-randomized controlled trials), 1, 2 (low quality studies), 3, 5 (high quality studies).

### Statistical analysis

The outcomes relevant for this meta-analysis were PFS, OS, ORR and toxicity. PFS was defined by the period from random assignment to the first documented disease progression. OS was defined by the period from random assignment to death from any cause, censoring patients who had not died at the date last known alive. ORR was defined as the sum of partial and complete response rates (according to the Response Evaluation Criteria in Solid Tumors (RECIST) (Therasse et al. 2000). Toxicity was graded according to the Common Toxicity Criteria version 2 (http://ctep.cancer.gov). The overall HRs for OS and PFS, the odds ratios (ORs) for ORR and AEs were calculated using statistical software (Stata version 13.0). Efficacy analysis was based on the intent-to-treat population, which was defined as all randomly assigned patients. All patients who received at least one dose of the study drug were included in safety analysis. A p-value less than 0.05 was considered significant. An HR > 1 reflects a favorable outcome in the Tax-PF arm for response rate while an OR > 1 indicates better overall response rates or higher toxicity in the Tax-PF arm. Both the fixed-effects model and the random-effects model was used to calculate the pooled estimates of efficacy. The quantification of the heterogeneity was calculated by the Cochrane *Q* statistic and the *I*^*2*^ value. The assumption of homogeneity was deemed invalid, when a p-value was < 0.1. Then the random-effects models were used after exploring the causes of heterogeneity. Otherwise, the fixed-effects models were used. The results of this meta-analysis were presented by forest plots. The individual squares represent each study’s HR or OR estimate while the lines extending from the squares represent the 95% confidence interval (CI) for the estimate. The size of the square represents the weight that the corresponding study exerts in the meta-analysis. In addition, the funnel plot, in which the standard error of log (OR) of each study was plotted against its log (OR) and Begg’s and the Egger’s linear regression test were used to estimate the publication bias. The significance of the intercept was displayed using the t-test suggested by Begg (p < 0.05 was considered as statistically significant) (Egger et al. 1997) (see Additional file 1: Figure S1).

## Results

### Description of included trials

A total of 1098 potentially relevant papers or abstracts were initially retrieved from the databases, of which 1091 could be excluded after thorough screening. A flow chart summarizing search results and exclusion strategy is provided in Figure 1. Finally, seven clinical trials were selected for the meta-analysis (Hitt et al. 2005; Posner et al. 2007; Vermorken et al. 2007; Pointreau et al. 2009; Vermorken et al. 2011; Lorch et al. 2011; Hitt et al. 2014).The main characteristics and detailed induction chemotherapy regimens are summarized in Table 1.

**Table 1:** **Characteristics of randomized controlled clinical trials in the meta-analysis**

**First author**	**Year**	**Study**	**NCT number**	**Center**	**Inclusion period**	**Total number**	**TPF/PF**	**Follow-up mean (month)**	**Induction Chemotherapy (IC)**	**Treatment after IC**	**Performance status**	**Median age(year)**	**Male (%)**	**Unresectable (%)**	**Jaded score**
Hitt	2005	/	/	SP	1998-2001	382	189/193	23.2	paclitaxel+PF vs PF	chemoradiotherapy	ECOG 0-1	TPF 56/PF 55	TPF 94/PF 94	TPF 64/PF 66	4
Hitt	2014	TTCC	NCT00261703	SP	2002-2007	439	155/156	23.8/22.1	docetaxel+PF vs PF	chemoradiotherapy	ECOG 0-1	TPF 58/PF 58	TPF 94/PF 93	100	4
Lorch	2011	TAX324	NCT00273546	US	1999-2003	501	255/246	72.2	docetaxel+PF vs PF	chemoradiotherapy	WHO-PS 0-1	TPF 55/PF 56	TPF 84/PF 83	TPF 67/PF 64	4
Posner	2007	TAX324	NCT00273546	US	1999-2003	501	255/246	42	docetaxel+PF vs PF	chemoradiotherapy	WHO-PS 0-1	TPF 55/PF 56	TPF 84/PF 83	TPF 67/PF 64	4
Vermorken	2007	EORTC 24971/TAX323	NCT00003888	BL	1999-2002	358	177/181	32.5	docetaxel+PF vs PF	radiotherapy	WHO-PS 0-1	TPF 53/PF 53	TPF 90/PF 90	100	4
Vermorken	2011	EORTC 24971/TAX323	NCT00003888	BL	/	308	156/152	103.2	docetaxel+PF vs PF	radiotherapy	/	/	/	100	3
Pointreau	2009	GORTEC	NCT00169182	FR	2000-2005	213	110/103	36	docetaxel+PF vs PF	radiotherapy and/or chemoradiotherapy	Karnofsky PS 100-80	TPF 57/PF 56	TPF 92/PF 94	/	4

### Three-year efficacy rate

**Figure 2:**
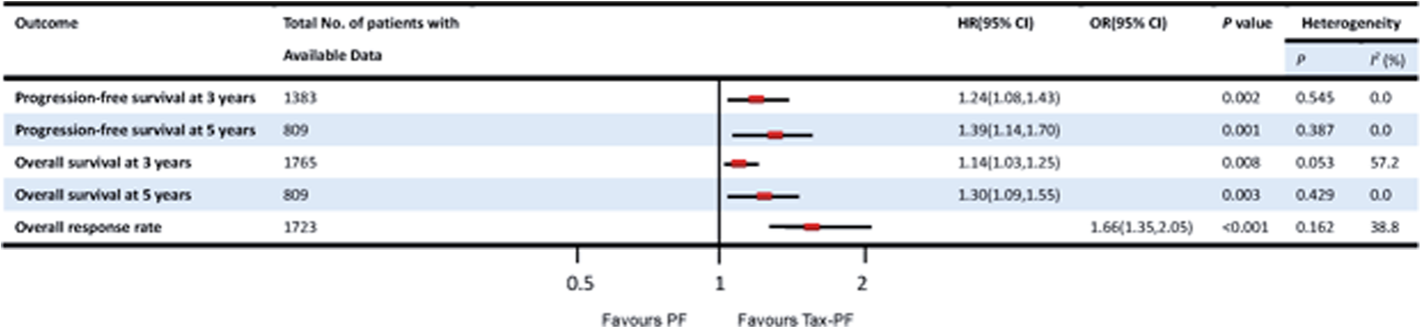
Clinical outcome after three 3 and 5 years.

Four trials (Posner et al. 2007; Vermorken et al. 2007; Pointreau et al. 2009; Hitt et al. 2014) provided data regarding the 3-year PFS rate. When combined, the data from the four trials yielded an estimated common HR of 1.24 (95% CI: 1.08 to 1.43). A significantly positive effect on survival was found for the use of Tax-PF for induction chemotherapy (*P* =0.002). Five trials (Hitt et al. 2005; Posner et al. 2007; Vermorken et al. 2007; Pointreau et al. 2009; Hitt et al. 2014) provided data on the 3-year OR rate. Patients treated with the Tax-PF regimen had a significantly longer 3-year OR rate (HR: 1.14; 95% CI: 1.03 to 1.25; *P* = 0.008). However, a between-trial-heterogeneity was observed for the OS analysis with an *I*^*2*^ value of 57.2% (*P* = 0.053), but not in PFS (*P* = 0.545). This heterogeneity was mainly related to the Spanish Head and Neck Cancer Cooperative Group (TTCC) in 2014 (Hitt et al. 2014). After eliminating this study, a reduction in the hazard of progression for Tax-PF group (HR 1.19, 95% CI 1.07 to 1.32, heterogeneity *P* = 0.132, *I*^*2*^ = 46.5%) was found. The main study results are detailed in Figure 2.

### Five-year efficacy rate

Of the four eligible studies, 2 trials (Vermorken et al. 2011; Lorch et al. 2011) were a long-term update to previous trials. From these studies, we could extract data to calculate the five-year efficacy rate for this meta-analysis. Tax-PF induction chemotherapy improved the PFS rate when compared with PF induction chemotherapy, with an HR of death of 1.39 (95% CI, 1.14 to 1.70; *P* = 0.001; Figure 2). Tax-PF induction chemotherapy also improved the OS rate, with an HR of 1.30 (95% CI, 1.09 to 1.55; *P* = 0.003). The test for heterogeneity of the data yielded a p-value > 0.1 (PFS: 0.387 and OS: 0.429); thus, we accepted the hypothesis of homogeneity.

### Overall response rate

The data of five trials (Hitt et al. 2005; Posner et al. 2007; Vermorken et al. 2007; Pointreau et al. 2009; Hitt et al. 2014) were included to determine the overall response (WHO criteria) to chemotherapy. Patients treated with Tax-PF had a significantly higher ORR (OR 1.66; 95% CI, 1.35 to 2.05; *P* < 0.001). No significant heterogeneity was found among studies used for analysis (heterogeneity *P* = 0.162, *I*^*2*^ = 38.8%).

### Toxicity

**Figure 3:**
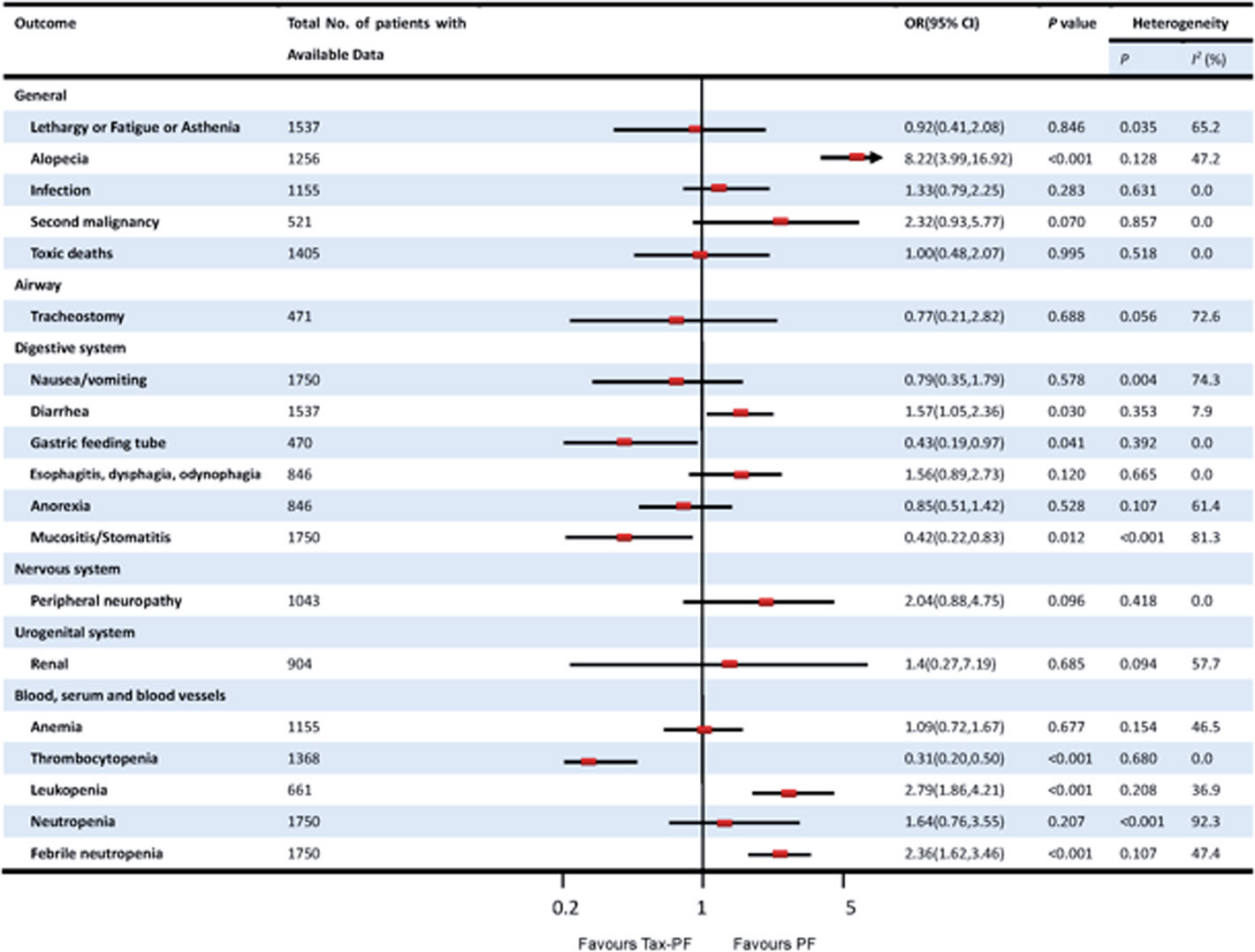
Toxicity profile of Tax-PF and PF regimen.

Data to analyse adverse events could be extracted from seven trials. The results are presented in Figure 3. Patients treated with the Tax-PF regimen had a significantly higher occurrence of grade 3 to 4 febrile neutropenia (OR 2.36; 95% CI, 1.62 to 3.46; *P* < 0.001), alopecia (OR 8.22; 95% CI, 3.99 to 16.92; *P* < 0.001), diarrhea (OR 1.57; 95% CI, 1.05 to 2.36; *P* = 0.03) and leukopenia (OR 2.79; 95% CI, 1.86 to 4.21; *P* < 0.001). Heterogeneity was found for some adverse events, which may be attributed to patient characteristics and to the use of different agents at various dosages in the studies.

### Publication bias

Egger’s test was performed to determine the publication bias of the literature. The results of Egger’s test did not suggest any evidence of publication bias (*P* = 0.854) (see Additional file 1: Figure S1).

## Discussion

Taxanes are a group of anticancer drugs that function by disruption of microtubule assembly and -function and can effectively block the cell cycle in G2/M phase which results in a mitotic arrest (Schrijvers and Vermorken 2000). In vitro studies suggested that taxanes also may play a role in radiation sensitization (Cui et al. 2014). Since the 1990s, taxanes have been evaluated for their potential to treat HNSCC (Qin et al. 2012).

The MACH-NC Collaborative Group performed an individual patient data meta-analysis with an inclusion of 1772 patients that were recruited between 1998 and 2007. Patients treated with Tax-PF had the benefit of a higher rate of loco-regional control (HR, 0.79; 95% CI, 0.66–0.97; *P* = 0.007), suffered from distant failures less frequently (HR, 0.63; 95% CI, 0.45–0.89; p = 0.009) and showed an absolute decrease in mortality at 5 years of 9.3% (PF vs TPF: 60.1% vs 50.8%). Nevertheless, the trials displayed heterogeneity regarding criteria such as patient inclusion, patient characteristics, drug regimens, tumor site, treatment intent, primary endpoint, and especially, definitive local treatment (Forastiere et al. 2013). The individual trial results also diminished the importance of the meta-analysis (Mak and Glisson 2014). The outcome of induction chemotherapy with a three-drug-combination consisting Tax-PF for locally advanced head and neck cancer is still a subject of debate and will remain so for the near future.

This meta-analysis evaluated not only the 3-year efficacy and safety, but also demonstrated for the first time a significant improvement in 5-year efficacy and safety of Tax-PF from two clinical studies. The long-term results from the other three trials (Hitt et al. 2014; Hitt et al. 2005; Pointreau et al. 2009) are not available at present. Therefore, we conclude at this point in time that the induction chemotherapy regimen using Tax-PF is superior to the PF regimen in terms of efficacy according to the currently available data. However, the long-term results of the currently ongoing trials should be included whenever available to increase the validity of this statement.

With regards to toxicities, the Tax-PF regimen led to a higher rate of grade 3 to 4 febrile neutropenia, alopecia, diarrhea and leucopenia as it has been shown in this meta-analysis (Figure 3). No difference was seen in toxicity-related mortality, implying that there was no increase in treatment-related deaths with Tax-PF treatment as compared with PF-regimen alone. The incidences of lethargy, infection, nausea and vomiting, peripheral neuropathy, renal function and anemia were not significantly different.

Severe adverse effects seemed predictable and manageable. Taking into account the toxicity-profile of Tax-PF-induction chemotherapy on one hand and the improved PFS and OS on the other future studies should aim to identify characteristics of treated patients that will help to determine the risk and benefit of distinct groups. One currently proposed strategy is a stratification of trial-results based on the distinct etiologies of HNSCC in future research since human papillomavirus (HPV)- and tobacco-related HNSCC exist (Qian et al. 2014; Qian et al. 2013). Fakhry et al. observed the HPV status of oropharyngeal HNSCC in a prospective clinical trial and confirmed, that tumor HPV status is strongly associated with therapeutic response and survival (Fakhry et al. 2008). Patients with HPV-positive tumors displayed higher response rates to induction chemotherapy including intravenous paclitaxel (82% vs 55%, *P* = 0.01) and after chemoradiation treatment (84% vs 57%, *P* = 0.007) than those with HPV-negative tumors. After a median follow-up of 39.1 months, compared with patients with HPV-negative tumors, patients with HPV-positive tumors demonstrated a significant improved OS (2-year OS = 95% vs 62%, difference = 33%, 95% CI = 18.6% to 47.4%, P = 0.005, log-rank test). Consequently, de-escalation treatment protocols of HPV-associated HNSCC were developed (Masterson et al. 2014). However, it will be necessary and of clinical interest to identify further the underlying mechanisms that determine a response to induction chemotherapy in patients with HPV-associated HNSCC and non-HPV-associated HNSCC to facilitate a proper selection of patients that are most likely to benefit from this therapy and thereby justifying the relatively high risk of therapy-induced toxicity. These approaches may include reverse bedside-to-bench research to stratify the influence of patient’ habits and biological factors on therapeutic outcome.

As stated by Forastiere et al. and others the ideal sequence of chemotherapy, radiation, and surgery for the management of loco-regionally advanced HNSCC has not been finally defined yet (Forastiere et al. 2013; Argiris et al. 2008).

Although non-surgical standard treatment of locally advanced HNSCC is concurrent chemoradio-therapy (Pignon et al. 2009; Petrelli et al. 2014; Masterson et al. 2014), there is emerging evidence, being now further consolidated and explored, that treatment with a three-drug regimen such as Tax-PF followed by surgery and consolidation chemoradio-therapy of patients with recurrent advanced HNSCC improved the response rates and survival (Yang et al. 2014). Other groups also reported that induction chemotherapy tended to improve clinical outcome with manageable toxicity (Won et al. 2014). Also the choice of the chemotherapeutic agent in a non-induction setting is not without controversy. While some authors view platinum-based chemoradio-therapy as the treatment of choice for locally advanced HNSCC (Petrelli et al. 2014; Masterson et al. 2014) and superior to radiotherapy combined with cetuximab, which should therefore be reserved for cases where the application of platinum-based agents is contraindicated, others recently argued that an improved OS and reduced toxicity supports the choice of taxane-based regimens in a concurrent setting over platinum non-taxane containing regimen (Behera et al. 2014). However, the findings also indicated that a better standardization of a taxane-based regimen is needed (Behera et al. 2014).

Some shortcomings of this analysis should be taken into account: First, like every meta-analysis, the quality of the results is limited by the quality of the included trials. Second, treatments were involved in a few trials based on the different dosages. Third, only seven RCTs were included and not all articles contained data of OS, PFS, ORR, and some adverse effects. Fourth, although publication bias was not found, the influence of the omission of further ongoing or yet unpublished studies cannot be included at the time of the preparation of this manuscript. However more long-term evidence from randomized studies is needed to further validate the therapeutic efficacy of Tax-PF. Finally, heterogeneity of the data exists in some outcomes in this meta-analysis, for which differences in patient populations, concurrent chemoradio-therapies, lengths of treatment and tumor performance status across the included trials may be responsible.

In conclusion, our data demonstrate that the Tax-PF induction chemotherapy regimens lead to a significant survival advantage at the cost of an increased rate of toxicity-related adverse effects as compared to the PF regimen. Therefore, before administration of Tax-PF induction chemotherapy regimen, followed by radiotherapy or concurrent chemoradio-therapy, a careful selection of patients seems advisable to reduce the incidence and severity of adverse effects. Ideally, these patients should be included in clinical trials to provide more data for future analysis and perspectively for better clinical decision making. For future research, investigators should include markers in their studies that help to distinguish subsets of patients e.g. according to risk-profile and HPV association of the HNSCC to further characterize groups at high risk for adverse effects and those most likely to benefit.

## Additional file

Additional file 1: Figure S1. Publication bias.
