# Supplementary figures and images for: Taxane-cisplatin-fluorouracil as induction chemotherapy for advanced head and neck cancer: a meta-analysis of the 5-year efficacy and safety
Source: Springerplus. 2015 May 1;4:208. doi: 10.1186/s40064-015-0988-5 (PMC4422827; doi:10.1186/s40064-015-0988-5)

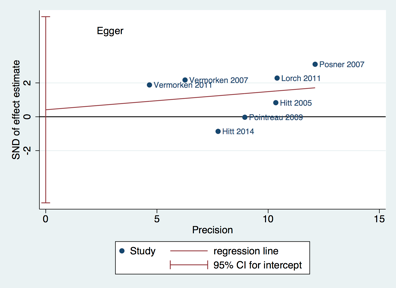

Supplement: Additional file 1: Figure S1. — Publication bias. [file 40064_2015_988_MOESM1_ESM.tiff]
